# Ischemic and Non-ischemic Stroke in Young Adults – A Look at Risk Factors and Outcome in a Developing Country

**DOI:** 10.7759/cureus.17079

**Published:** 2021-08-11

**Authors:** Mohammed Tahar Si Larbi, Waleed al Mangour, Iram Saba, Dhekra Al Naqeb, Zaina Swapna Faisal, Sana Omar, Fatima Ibrahim

**Affiliations:** 1 Medical Affairs, Sultan Bin Abdulaziz Humanitarian City, Riyadh, SAU; 2 Research, Sultan Bin Abdulaziz Rehabilitation Center Riyadh, Riyadh, SAU; 3 Research and Scientific Center, Sultan Bin Abdulaziz Humanitarian City, Riyadh, SAU

**Keywords:** young adults, ischemic stroke, risk factors, rehabilitation, functional independence measure (fim)

## Abstract

Objective

Stroke among young adults is the leading cause of disability worldwide. Efforts are being taken to control stroke in the general population, but in parallel, there is an increasing trend of stroke among the young population. These patients are often affected by physical disability, cognitive impairment, and loss of productivity, all of which have personal, social, and economic implications. The main aim of this study was to determine the risk factors associated with stroke among young patients admitted to a tertiary care rehabilitation center and determine the effect of rehabilitation on the outcome of their daily life activities.

Materials and Methods

A retrospective hospital-based cohort study was conducted between January 2015 to December 2019. Prevalence of stroke-related risk factors like hypertension, hyperlipidemia, diabetes, and cardiac disease was assessed.

Results

Out of 710 young stroke adults, 71.97% were described as ischemic, and 28.03% reported as non-ischemic. Mean age (SD) was found to be 44.54 ± 9.3. Univariate analysis demonstrated that hyperlipidemia, cardiac disease, and diabetes indicated a significantly higher risk for ischemic stroke with an OR (95% CI) at 2.5 (1.7-3.7), 2.11 (1.2-3.6), and 1.66 (1.2-2.3) respectively. A significant improvement was observed in their Functional Independence Measure (FIM0 score after their rehabilitation irrespective of age and gender.

Conclusion

Association of risk factors associated with stroke should be subjected to close follow-up and management, thus reducing the risk of developing long-lasting disabilities at a young age. The identification of risk factors for young stroke incidence is a step towards improving health in the young adult population.

## Introduction

Stroke is a common neurological disorder increasing worldwide with 15 million strokes and 5.8 million stroke-related deaths per year [1]. The majority of incident cases of stroke are ischemic stroke (87%), followed by intracerebral hemorrhage (10%) and subarachnoid hemorrhage (3%) [2]. Stroke in young adults is more heterogeneous than stroke in older persons, owing to the wide range of underlying risk factors and etiology. Despite an increasing number of young stroke patients, the risk factors of stroke remain unknown in about one-third of all patients [3]. The disturbing patterns of stroke in young adults are likely due to growing rates of modifiable risk factors such as hypertension, hyperlipidemia, obesity, and diabetes, highlighting the importance of early detection and active prevention measures in the general population at an early age [4]. Stroke among young patients has special attention since they have shown better recovery. This is different from stroke seen in developed countries, where a majority of stroke cases affect the older population. 

The stroke specialty program of Sultan-Bin-Abdul-Aziz Humanitarian City (SBAHC), Riyadh, Saudi Arabia, provides individualized patient-centered, coordinated, and integrated services. The program had focused on different age-specific factors such as pregnancy, oral contraceptive use, and behavioral factor of the young population such as low physical activity and smoking. In developing countries, there is a lack of data on stroke among young patients despite such problems are rapidly growing; however, research in such areas is essential to plan for appropriate screening, diagnosis, and management. It is difficult and often subjective to identify an age cut-off, but young adults are usually identified as those under 55 years old in previously published studies and registries [5].

This retrospective hospital-based study aims to assess the different stroke types and risk factors of stroke in young patients and to assess post-stroke complications, outcomes, and activities of daily living using the Functional Independent Measure (FIM) instrument.

## Materials and methods

Study population

This is a retrospective hospital-based cohort study for patients diagnosed with stroke admitted to the stroke unit at Sultan bin Abdul Aziz Humanitarian city (SBAHC) for a rehabilitation program between January 2015 to December 2019. The inclusion criteria included the patients having a history of stroke for at least 6 months and aged between 18 to 55 years for both genders. The patients with a history of intracranial congenital diseases, malignancy, neurological diseases, and psychiatric illnesses were excluded. All the involved cases were classified as per the International Classification of Diseases, Tenth Revision (ICD-10) [6]. Ischemic stroke is an episode of neurological dysfunction caused by focal cerebral, spinal, or retinal infarction. Intracerebral hemorrhage is a focal collection of blood within the brain parenchyma or ventricular system that is not caused by trauma, while stroke, which is not otherwise specified, is defined as an episode of acute neurological dysfunction presumed to be caused by ischemia or hemorrhage, persisting for ≥24 hours or until death.

Data collection

The data was collected using the case report form (CRF) that was pre-tested for the feasibility of the data collection process. The CRF was completed with the socio-demographic data (age, gender, and residence area), clinical assessment history of diseases (such as ischemic non-ischemic, hypertension, hyperlipidemia, cardiac disease, and family history of hypertension and diabetes) were also included. Smoking history was collected. Laboratory data include hemoglobin, white blood cells (WBC), and platelets in addition to metabolic markers, i.e., fasting and random blood sugar, HbA1c, lipid profile, including total cholesterol, low-density lipoprotein (LDL), and low-density lipoprotein (HDL) in addition to urea, creatinine, Na, and K.

To assess the risk factors of stroke in young patients and post-stroke complications and outcomes in activities of daily life, a Functional independent measurement (FIM) [7] instrument was used. The FIM Score is a 126-point instrument that comprises 18 individual subscales measuring a variety of physical and cognitive functions. Each subscale is scored from 1 to 7 (1 = total assist, 7 = complete independence), resulting in a total FIM score that ranges from 18 to 126. The FIM is well known and validated in the rehabilitation and stroke populations. Permission to use the FIM in this study was granted under the Medical Rehabilitation program of SBAHC. The study was approved by the Institutional Review Board of Sultan bin Abdul Aziz Humanitarian city (SBAHC), Riyadh, Kingdom of Saudi Arabia.

Statistical Analysis

The chi-square test and Mann-Whitney U test were used to analyze the categorical and continuous data respectively. The relationship between risk factors and stroke type was examined by univariate and age-gender adjusted logistic regression. Odds ratios (with 95% confidence interval) were used for assessing stroke risk factors using the result of univariate analysis, while age and gender-adjusted models for logistic regression analysis were used to control any confounders. Statistically significant was consistent if p-value <0.05.

## Results

Characteristics of the study population

The study included 710 young stroke patients (18- 55 years) who fulfill the criteria of stroke and have been assessed for risk factors. Out of the total number, 511 (71.97%) were described as an ischemic type, and 199 (28.03%) were reported as non-ischemic type (hemorrhage or others). Table 1 demonstrates the demographic characteristics for 480 (67.6%) male and 230 (32.4%) females with mean age (SD) of 44.74 (9.39) and 44.03 (8.95) respectively.

**Table 1:** Study cohort clinical, hematological, metabolic, and biochemical characteristics Note: Data Presented as Mean ± SD and Median (1st Q-3rd Q) for Gaussian and Non-Gaussian variables. P-value was calculated for differences between the two groups, and p<0.05 was considered as significant. WBC - white blood cells, LDL - low-density lipoprotein, HDL - high-density lipoprotein

Variables	All	Ischemic stroke	Non-ischemic stroke	P-value
Number	710	511 (71.97)	199 (28.03)	
Age (years)	44.54 ± 9.27	44.74 ± 9.39	44.03 ± 8.95	0.35
Male	480 (67.6)	347 (72.3)	133 (27.7)	0.79
Female	230 (32.4)	164 (71.3)	66 (28.7)	0.78
Currently employed	111 (41.3)	77 (69.4)	34 (30.6)	0.56
Currently unemployed	158 (58.7)	109 (69.0)	49 (31.0)	0.366
Living in urban area	550 (77.5)	395 (71.8)	155 (28.2)	0.92
Living in rural area	160 (22.5)	116 (72.5)	44 (27.5)	0.92
Smoking	94 (4.1)	67 (71.3)	27 (28.7)	0.90
Weight (kg)	76.16 ± 17.23	75.90 ± 16.87	76.81± 18.16	0.51
Height (cm)	164.63 ± 13.54	164.52 ± 14.68	164.92 ± 10.04	0.64
BMI(kg/m^2^)	23.16 ± 5.07	23.11 ± 5.06	23.28 ± 5.11	0.69
Systolic blood pressure	120.46 ± 17.48	120.49 ± 17.99	120.38 ± 16.1	0.94
Diastolic blood pressure	75.7 ± 9.66	75.44 ± 9.56	76.38 ± 9.91	0.24
Hypertension	442 (62.3)	308 (69.7)	134 (30.3)	0.085
Diabetes	308 (43.3)	239 (77.6)	69 (22.4)	<0.001
Hyperlipidemia	247 (34.7)	205 (83.0)	42 (17.0)	0.003
Cardiac disease	112 (15.8)	93 (83.0)	19 (17.0)	0.004
Blood hemoglobin	13.3 ± 6.3	13.3 ± 7.06	13.11 ± 3.76	0.60
WBC	6.83(5.5 - 8.6)	6.85(5.5 - 8.8)	6.8(5.5 - 8.3)	0.034
Platelets	264 (224 - 321)	264 (228 - 224)	260(219 - 314)	0.096
Fasting blood sugar	5.37 (3.1 - 6.3)	5.4 (4.9 - 6.65)	5.3 (4.86 - 5.94)	0.49
Random blood sugar	6.1 (5 - 9.4)	6.5 (5.17 - 9.8)	5.6 (4.9 - 7.6)	0.95
HbA1c	5.9 (5.3 - 7.2)	6.1 (5.4 - 7.4)	5.5 (5.2 - 6.3)	0.60
Urea	5.3 ± 2.8	5.16 ± 2.7	5.53 ± 3.06	0.22
Serum creatinine	66.6 (54 - 80)	66.4 (54.3 - 80.4)	67 (55.9 - 82.8)	0.50
Serum sodium	141.69 ± 58.8	142.53 ± 64.8	139.4 ± 38.79	0.49
Serum potassium	4.03 ± 0.84	4.05 ± 0.75	3.97 ± 1.03	0.07
Total cholesterol	4.02 ± 1.1	3.94 ± 1.1	4.22 ± 1.0	0.002
LDL	2.27 ± 1	2.19 ± 1	2.47 ± 0.9	0.32
HDL	1.08 ± 0.3	1.07 ± 0.3	1.1 ± 0.3	0.82
Triglycerides	1.37 (1-1.8)	1.38 (1-1.8)	1.36 (1-1.8)	0.86

The study cohort did not demonstrate any clinical, biochemical, and hematological difference between ischemic and non-ischemic patients. Further analysis indicated that the proportion of patients suffering from diabetes, hyperlipidemia, and cardiac diseases were significantly higher in Ischemic vs. non-ischemic stroke (p-values of <0.001, 0.003, and 0.004, respectively). Total cholesterol levels were significantly lower among ischemic stroke (3.94 ± 1.1 l mmol/l vs. 4.22 ± 1.0 mmol/l, p=0.002).

**Figure 1:**
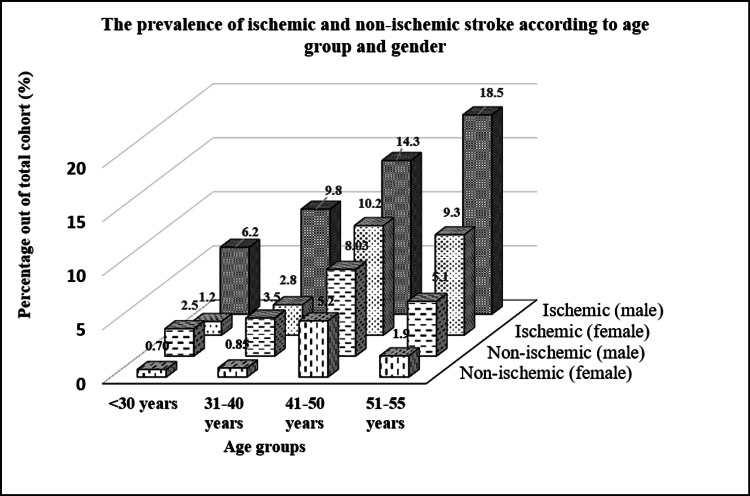
Distribution for Age and Gender in relation to stroke type P-value was significant (<0.001) for each age group between Ischemic and non-ischemic patients except for group <30 years in females (p=0.91).

The prevalence of stroke increases with age regardless of type or gender except for the age group > 50 years for non-ischemic stroke in both genders and ischemic female group. There are more male stroke patients than females in any given age group. Figure 1 shows the highest stroke prevalence was among ischemic males aged > 50 years, while the lowest was observed among non-ischemic females aged < 50 years.

Complications associated with stroke in the study population

Analysis of the relationship between stroke-associated complications with that of gender, age, and etiology was presented in Table 2.

**Table 2:** Distribution of complications associated with stroke in relation to age, gender, and types of strokes Note: *Physical disabilities: mobility, speech, swallowing disabilities. **Other complications: pulmonary embolism, thyroid disorder, bipolar syndrome, kidney disorder, anemia, cardiomyopathy.

Variable	Total (%)	Gender			Age			Etiology		
		Male	Female	Pivalue	<30	≥30	Pivalue	Ischemic	Non-ischemic	P-value
Seizure	90 (12.70)	58(64.40)	32(35.60)	<0.001	21(23.30)	69(76.70)	<0.001	60(66.60)	30(33.40)	<0.001
Speech impairment	90 (12.70)	55(61.10)	35(38.90)	0.0028	23(25.50)	67(74.50)	<0.001	64(71.20)	26(28.80)	<0.001
Depression	52 (7.30)	36(69.24)	16(30.76)	<0.001	20(38.40)	32(61.60)	0.019	34(65.30)	18(34.70)	<0.001
Urinary Incontinence	46 (6.50)	32(69.56)	14(30.44)	<0.001	12(26.80)	34(73.20)	<0.001	29(63.10)	17(36.90)	0.01
Recurrent Stroke	40 (5.60)	25(62.50)	15(37.50)	0.02	7(17.50)	33(82.5)	<0.001	29(72.50)	11(27.50)	<0.001
Constipation	28 (3.90)	22(78.57)	6(21.43)	<0.001	15(53.70)	13(46.30)	<0.001	19(67.80)	9(32.20)	<0.001
*Physical disabilities	154(21.7)	101 (65.60)	53(34.40)	<0.001	35(22.72)	119(77.28)	<0.001	110(71.42)	44(28.58)	<0.001
**Other complications	60(8.30)	42(70.00)	18(30.00)	<0.001	8(13.34)	52(86.66)	<0.001	40(66.67)	20(33.33)	0.0003

Physical disabilities were found in 21.7% affecting more males older than 30 years mainly ischemic in nature. The second most frequent complications were seizures and speech impairment at the rate of 12.70%, which is significantly higher in males at an age older than 30 years and ischemic in etiology. The other complications were depression and urinary incontinence at the rate of 7.3% and 6.5%, respectively.

The stroke recurrence was found to be at the rate of 5.6% in this cohort and was more among males who were older than 30 years of age and had an ischemic stroke. The other associated complications, including mobility, speech, swallowing, and other impairments, were also significantly higher in ischemic male patients who were above ≥30 of age. 

FIM scores before and after rehabilitation

Table 3 demonstrates a significant increase in Functional Independent Score (FIM) score at discharge when compared with admission in genders, different age group, etiology, and pre-morbidity.

**Table 3:** FIM score before and after rehabilitation program for the total cohort. Note: P-value between different gender, etiology, and pre- morbidity were non-significant, while p-value was significant for age-group between FIM admission and Discharge Score. FIM - Functional Independent Measure

Risk factors		FIM Score Mean ± SD		
		Admission score	Discharge score	P-value
Gender	Male	77.63 ± 27.74	94.28 ± 25.96	<0.001
	Female	77.7 ± 27.71	94.36 ± 25.99	<0.001
Age	<30 years	87.09 ± 22.48	101.28 ± 20.15	0.0001
	>30 years	76.57 ± 28.04	93.50 ± 26.42	<0.001
Etiology	Ischemic	77.72 ± 27.69	94.37 ± 25.97	<0.001
	Non-Ischemic	77.45 ± 27.45	94.17 ± 26.02	<0.001
Pre-Morbidity	Hypertension	74.31 ± 27.80	94.30 ± 26.00	<0.001
	Diabetes mellitus	72.44 ± 28.00	89.38 ± 27.30	<0.001
	Hyperlipidemia	76.92 ± 26.70	93.33 ± 24.92	<0.001
	Cardiac Disease	73.77 ± 28.07	91.01 ± 26.46	<0.001

Although there was no significant difference within those subgroups either during admission or discharge, a significant difference was found between FIM admission and FIM discharge in these groups.

The adjusted odds ratio for important risk factors among the studied cohort

The odds ratio for important risk factors has been given in Table 4. 

**Table 4:** Univariate and age-gender adjusted odds ratio and confidence interval (95% CI) for important risk factors among studied cohort

	Univariate					Age and gender adjusted		
Risk	Ischemic (511)	Non-Ischemic (199)	OR	CI	P-value	OR	CI	P-value
Hyperlipidemia	205 (40.1)	42 (21.1)	2.50	1.7 - 3.7	<0.001	2.54	1.7-3.7	<0.001
Cardiac disease	93 (18.2)	19 (9.5)	2.11	1.2 - 3.6	0.005	2.15	1.3-3.6	0.004
Diabetes	239 (46.8)	69 (34.7)	1.66	1.2 - 2.3	0.004	1.64	1.2-2.3	0.005
Hypertension	308 (60.3)	134 (67.3)	0.74	0.5 - 1.1	0.08	0.73	0.5-1.1	0.07
Male gender	347 (67.9)	133 (66.8)	1.05	0.7 - 1.5	0.78	-	-	-
Age ≥ 45	308 (60.27)	113 (56.78)	1.15	0.82 – 1.6	0.39	-	-	-

The univariate analysis in Table 4 demonstrated that hyperlipidemia, cardiac disease, and diabetes indicated a significantly higher risk for Ischemic stroke with OR (95% CI) at 2.5 (1.7-3.7), 2.11 (1.2 - 3.6), and 1.66 (1.2 - 2.3) respectively. Hypertension, male gender, and age ≥ 45 years remained as second most important risk factor, with OR (95% CI) at 0.74 (0.5 - 1.11), 1.05 (0.7 - 1.5) and 1.15 (0.82 - 1.6) respectively. hyperlipidemia, cardiac disease, and diabetes were also significant risk factors in age and gender-adjusted regression analyses.

## Discussion

This large cohort study over a long period collected young stroked patients from wide areas in the Kingdom, being a tertiary rehabilitation center. In developing countries mean age of the first stroke is 63 years, compared to 69 in the US and 70 in England, which indicates that developing countries are hosting more young populations with stroke [8]. Although we are presenting non-acute stroke patients, this study had brought up a better understanding for patients suffering from ischemic stroke versus non-ischemic stroke in the young age group (18-55 years). Prevalence of stroke regardless of etiology demonstrates increment with age although males are three times more than females for ischemic stroke in different age groups, which is an observation reported from Helsinki Young Stroke Registry for age-specific stroke [9]. This could be related to the low prevalence of vascular changes observed in females based on their hormonal factors, in addition to the fact that males are prone to large vessel atherosclerosis at this age [10].

Non-ischemic stroke was more predominant among males, especially below 40 years, mainly in the form of a subarachnoid and cerebral hemorrhage. This is the same observation as found in an inter-stroke study that has investigated different risk factors for stroke involving 32 countries, including data from the middle east [11]. As males are more prone to trauma than females, we can see a higher incidence of hypertension among them which later contribute to intracerebral and subarachnoid hemorrhage [12].Other explanations like a higher incidence of arteriovenous malformation prevalence in males when compared with females [13].

This study age spectrum was between 18 to 55 years where there is an increase in the prevalence by 50% with any 10 years increment of the age. The drop-in prevalence of non-ischemic stroke is most likely related to a decrease rate of risk factors associated with non-ischemic strokes like trauma, congenital anomalies, and malignant hypertension [14]. Each year almost 11 million ischemic strokes occur globally, half of which occur in low and middle-income countries [15]. A significant cause of long-term impairment in this condition has a profound influence on patients and caregivers on the quality of life.

Although hypertension is the most frequent risk factor in both ischemic and non-ischemic patients, the univariate and age/gender-adjusted odds ratio did not demonstrate this risk factor to be of a significant value that is similar to the findings from a retrospective hospital-based study from Iran [16]. This could result from antihypertensive drug therapy use, as well as lifestyle modification changes among young adults. A meta-analysis study conducted by Law et al. showed that when the systolic and diastolic blood pressure was reduced by antihypertensive therapy, the incidence of stroke declined by 41 % [17].

In the current study, we also found that hyperlipidemia is strongly associated with an ischemic stroke which is observed by Ansell et al., where they report the risk of ischemic stroke to be almost double that of non-ischemic stroke [18]. The Strong Heart Study revealed that greater lipoprotein cholesterol level confers a high risk of ischemic stroke. High triglycerides and low HDL levels observed in patients suffering from diabetes have a more than two times higher incidence of ischemic stroke [19]. Although this study showed a lower value for total cholesterol and LDL as a result of therapeutic intervention, this will not eliminate the role of this important risk factor. In patients with large artery disease or disease of small vessels, hyperlipidemia is more frequently observed, while it is less common in ischemic patients having cardiac embolism [20]. In stroke due to large artery disease or small vessel disease, hyperlipidemia increases the risk among older adults who are often to have large artery disease and small-vessel disease due to stroke.

The studies have shown that diabetes among young adults is associated with a higher risk of stroke, and it's disturbing that the incidence of diabetes in young adults is increasing [21]. This study finds a significant association between diabetes and incidence of stroke; also, the univariate age and gender-adjusted odds ratio showed significant importance, particularly the ischemic stroke. This is inconsistent with other studies where diabetes is the main risk factor following hypertension and has been identified as a significant independent variable of symptomatic recurrence in patients with first-ever stroke [22]. This would stress the fact that glycemic control of patients with diabetes carries an important role in preventing ischemic stroke [23].

The other risk factor like cardiovascular disease also showed a significant univariate and age/gender-adjusted odds ratio. This could result in atherosclerotic changes, especially in the left ventricle observed in both ischemic cardiac and cerebral events showing the frequency of ischemic stroke to be inversely proportional to left ventricular ejection fraction [24].

A total of 5.6 % of the studied cohort were found to have a recurrent stroke. The other studies also reported the frequency of recurrent stroke between 2% to 18% [25]. The other stroke complications like seizures, depression, urinary incontinence, and constipation were found significantly higher in older male ischemic patients. A history of smoking was not significant, which could be due to a small number of smokers, especially among women in this community. 

Obesity was not observed among stroked patients regardless of the type of stroke. This is inconsistent with the Northern Manhattan Study, which has concluded that abdominal obesity is an independent risk factor for ischemic stroke in all ethnic groups where BMI has no value on such findings [26].

FIM score is a very important tool for young stroke patients, not for their initial assessment but their degree of improvement. When looking at gender, age, etiology, and pre morbidity factors, there was a clear significant improvement which is expected in younger patients when compared with the older age group. Early intervention and treatment of patients with stroke not only decreases death but also decreases impairment and increases the enhancement of survival and independence [27].

Our study did not use the TOAST (Trial of Org 10172 in Acute Stroke Treatment) rating system to classify subtypes of acute ischemic stroke due to incompleteness to gather data. However, the above limitation does not compromise the essential data for case definition and major risk factors. The other limitation was that it excluded the ones having a history of intracranial congenital diseases, malignancy, neurological and psychiatric illness.

## Conclusions

The study concludes that risk factors such as hypertension, hyperlipidemia, cardiac disease, and diabetes were more associated with an ischemic stroke rather than non-ischemic stroke, and this calls for a prevention plan at the national level with a focus on various lifestyle factors, proper rehabilitation process for young adults to prevent stroke and recommendations to help diagnose stroke at a young age and improve care. We are also looking forward to the implementation of a population-based study of stroke in each region and making ties with regional and national stroke registries here in Saudi Arabia. The identification of risk factors for young stroke incidence is a step towards improving health in young adults. Various organizations have disseminated awareness and encouraged research in this area. However, a more comprehensive study with larger numbers and well-defined risk factors is needed before such programs can be established. These types of treatments could lead to better treatment and management, potentially reducing the impact and burden of ischemic stroke in the highly productive age group.
